# Molecular docking and simulation analysis of the FimH protein with secondary metabolites from the *Garcinia* species

**DOI:** 10.6026/97320630018076

**Published:** 2022-02-28

**Authors:** Abhijeeth S Badiger, K Jayadev, G Manu, Ranjith Raj, Avinash Ammanagi, Shrisha Naik Bajpe, Ramith Ramu

**Affiliations:** 1 Department of Biotechnology, Sri Dharmasthala Manjunatheshwara College, Ujire - 574240, Karnataka, India; 2 Department of PG studies in Biotechnology, Alva's College, Moodbidri - 574227, Karnataka, India; 3 Department of Pharmacology, JSS Medical College, JSS Academy of Higher Education and Research, Mysuru - 570015, Karnataka, India; 4 Department of PG studies in Microbiology and Biotechnology, Karnataka University, Dharwad - 580003, Karnataka, India; 5 Department of Biotechnology and Bioinformatics, School of Life Sciences, JSS Academy of Higher Education and Research, Mysuru-570015, Karnataka, India

**Keywords:** Molecular docking, simulation, FimH protein, secondary metabolites, *Garcinia*species

## Abstract

It is of interest to document the molecular docking and simulation analysis of the FimH protein with 10 secondary metabolites from Garcinia species in the context of drug discovery for Urinary tract infections (UTIs). We report the optimal binding features of flavonoids with the FimH protein for further consideration in drug discovery.

## Background:

Urinary tract infection (UTI) is the most common ailment that affects women. E.coli is the most common causative pathogen, accounting for 80% of all UTIs [1]. The adhesion protein FimH is responsible for the bacterial cell's adherence to the epithelial cells of the urinary system that leads to the colonization for infection [2]. This protein is a potential candidate for the development of drugs and vaccine [3]. Uroplakins are trans membrane proteins specific to the urothelium that acts as a receptor that facilitate the binding of theFimH to the urinary tract [4]. Garcinia genus trees are endemic to the states of Maharashtra, Kerala, and Karnataka and grow in the peninsular coastal regions of India, including the Western Ghats, Eastern Ghats, and North-eastern regions [5]. The secondary metabolites of the fruits as well as the leaves and other parts of the tree, are medicinally significant [6]. Different species of Garciniahave demonstrated therapeutic potential for the treatment of wounds, pain, and infections, antioxidant, antinociceptive, antitumoral, antimicrobial,anticancer, antiulcerogenic, antihistaminic, and many other [7,8]. Among the various species from the genus Garcinia, Garcinia cambogiahas traditionally identified properties for the treatment of urinary tract infections [9]. Flavonoids present within the cranberry blocked the FimH-mediated contact of uropathogens with the host cell bladder epithelium [10,11]. Therefore, it is of interest to document the molecular docking [12] analysis of the FimH protein with 10 flavonoids from cranberry in the context of Urinary tract infections (UTIs). (-) -Hydroxycitric acid, Garcinol, isogarcinol, xanthochymol, cyanidin 3-glucoside, cyanidin 3-sambubioside, isoxanthochymol, combogic acid, alpha mangostin, and beta mangostin were the ten primary metabolites chosen for the study because of their therapeutic potential [13].

## Materials and Methods:

### Sequence and structure data:

The 3-dimensional (3D) structures of the compounds ((-) -Hydroxycitric acid, Garcinol, isogarcinol, xanthochymol, cyanidin 3-glucoside, cyanidin 3-sambubioside, isoxanthochymol, combogic acid, alpha mangostin, and beta mangostin) were downloaded from PubChem (https://pubchem.ncbi.nlm.nih.gov/) databasesin SDF format. The crystal structure of αFimH from E.coli (PDB: 6GTX) (14) were obtained from protein data bank (https://www.rcsb.org/) which has a resolution of 2.50 Å [14].

### Molecular docking simulations:

Molecular docking was done using AutoDock Vina 1.1.2 to get a better understanding of the complex interaction and the conformational changes that takes place during complex formation within the binding cavity of protein. The pre-docking preparation was done based on Autodock4.2 protocol using Autodock Tools 1.5.6 by removing water molecules and hetero atoms which were present in the protein structure. Further, by adding polar hydrogens and merging non-polar hydrogen the protein structure was stabilized. The required Kollmann united and Gasteiger-Marsili empirical atomic partial charges which were predicted by the autodock tool were assigned to the structure. However, for the ligand Kollmann united and Gasteiger charges were kept default. Later, for the protein AD4 atom type were assigned. The binding cavity required to build the energy grid was predicted using CASTp 3.0 online server. The result was analysed based on the binding affinity, total number of non-bonding interactions, and their hydrogen bonds using Biovia Discovery Studio Visualizer 2021 [15-17].

### Molecular dynamics simulation:

The stability of the docked complex was studied using molecular dynamics simulations to understand their behavior and conformational changes at the structural level using GROMACS-2018.1. The MD simulation was run for 100ns using CHARMM27 force field parameter. Using gmx tool, protein topology file was created whereas for ligand topology Swiss Param server (https://www.swissparam.ch/) was used. The TIP3 water model was used further, solvent box was created with 10 Å distance. To equilibrate the system, counter ions such as Na+ and Cl- were introduced while maintaining the salt concentration at 0.15 M. The initial energy minimization was done using the steepest descent algorithm. Further, for system equilibration the NPT and NVT ensemble classes were employed with a 310K temperature and 1 bar pressure. For every 10 ps, the system's coordinates and energies were saved. Finally, using XMGRACE software the trajectories were analysed by plotting graph for Root Mean Square Deviation (RMSD), Root Mean Square Fluctuation (RMSF), Radius of Gyration (Rg) and SASA (Solvent Accessible Surface Area)and hydrogen bond [11,14-17].

### Binding free energy calculations:

To understand the energy formed during protein-ligand complex formation after the dynamic study, molecular mechanics/Poisson-Boltzmann Surface Area (MM-PBSA) technique was employed. The result obtained after the simulation was considered to calculate free-binding energy by using gmmpbsa programme. The main three factors used in determination of the binding free energy are molecular mechanical energy, polar and polar solvation energies. To compute the binding free energy, the MD trajectories of the last 50 ns were used with dt 1000 frames [14-16,18].

## Results and Discussion:

Women suffer more with UTI and the pathogens causing urinary tract infection are multiple drugs resistant and diabetic patients are more susceptible for UTI [1]. Hence there is a need for drug discovery. Molecular docking being a great platform for drug discovery, it helps in screening of potential candidates for drug development and discovery [19]. Secondary metabolites of Garcinia species have showed inhibitory effect against FimH. Especially garcinol a benzophenone present in Kokum can be considered as a potential agent against UTI. Further QSAR studies are required to confirm the mode of action and affinity of the ligand against FimH [20]. Cranberry is a fruit that is native to America that grows in marshes and currently it is being used as medicine against UTI. Kokum being a tropical fruit and several species of Garcinia are endemic to India and can be used as a potent drug against urinary tract infection. Currently Kokum being an underutilized fruit, it is just used for domestic purposes [21]. But recent studies are throwing light on Kokum as a potent drug for many ailments. According to the present study, bioactive compounds from Garcinia sp. can be considered for drug discovery and development against UTI as the plant contain flavonoids, anthocyanins and majorly Hydroxycitric acid (HCA), Garcinol and Cambogic acid as secondary metabolites. HCA and garcinol are currently the metabolites of interest which are being extensively studied [22,23]. Further studies on this aspect can convert an underutilized fruit into a local bio resource.

After the virtual screening, garcinol complex had the highest binding affinity of -7.9 Kcal per mol with total of 12 non-bonded interactions and three hydrogen bonds when compared with that for all the other compounds tested. The result obtained after docking study of all the compounds along with their binding affinity, total number of non-bonded interaction and total number of hydrogen bond is given below Table 1(see PDF). Further, the detailed list of garcinol complex amino-acid residues along with their binding interaction is given in Table 2(see PDF). Thepictorial representation of interaction is illustrated in Figure 1 (see PDF).

MD simulation was used to validate the docked garcinol bound FimHcomplex, The simulation was conducted to get a better understanding of the structural rearrangement of garcinol-FimH complex as well as the stability of the complex formed during docking process. The trajectories obtained after the MD simulation was analysed using graph. The pictorial representation of graphs for RMSD, RMSF, Rg, SASA, H-bond is given in Figure 2(see PDF). Based on the RMSD plot, it can be predicted that the complex and the protein shows similar pattern and both are within the range of 0.2-0.25 nm and attains stability at ∼30 ns with slight fluctuation at beginning. To future, understand the residual fluctuation and to compare the flexibility of each residue in the complex RMSF plot was analysed, based on the plot the fluctuation is seen within loop terminal for both garcinol and target protein. The Radius of gyration (Rg) plot for both complex as well as protein was found to be in range ∼2.4 nmand were found to be stable with slight fluctuation, the similar result was seen for SASA plot analysis with the average value of both the garcinol complex and protein FimH showed 235 nm2, respectively. Based on the hydrogen bond analysis it can be predicted that there may be structural re-agreement taken place during MD simulation as the number of hydrogen bond as increased when compared to docked process, thus it can be predicted that garcinol complex has better stability.

The energy formed during complex formation was calculated using MM-PBSA method. The predicted value by calculation of binding free energy is given in Table 3(see PDF), which gives the detailed results of energy formedfor the duration for the last 50 ns. Based on the estimated value it can be said that the garcinol complex shows better stability has it has more negative values.Overall, the flavonoids present in the cranberry extract, especially garcinol demonstrated greatest interaction with the FimH protein indicating its ability to inhibit the colonization of the pathogen on the urinary tract. Further in vitro and in vivo studies on these lines will provide a strong basis in establishing this as a potential treatment for UTI.

## Conclusion:

We report the optimal binding features of secondary metabolites with the FimH protein with stability of the ligand-protein complex for further consideration in drug discovery.
